# A review of canine babesiosis: the European perspective

**DOI:** 10.1186/s13071-016-1596-0

**Published:** 2016-06-11

**Authors:** Laia Solano-Gallego, Ángel Sainz, Xavier Roura, Agustín Estrada-Peña, Guadalupe Miró

**Affiliations:** Department of Animal Medicine and Surgery, Faculty of Veterinary Medicine, Universitat Autònoma de Barcelona, Barcelona, Spain; Department of Animal Medicine and Surgery, Veterinary Clinic Hospital, Faculty of Veterinary Medicine, Universidad Complutense de Madrid, Madrid, Spain; Hospital Clínic Veterinari, Universitat Autònoma de Barcelona, Barcelona, Spain; Department of Animal Pathology, Faculty of Veterinary Medicine, University of Zaragoza, Zaragoza, Spain; Department of Animal Health, Veterinary Clinic Hospital, Faculty of Veterinary Medicine, Universidad Complutense de Madrid, Madrid, Spain

**Keywords:** Babesiosis, Canine, *Babesia*, Guideline, Consensus

## Abstract

Canine babesiosis is a significant tick-borne disease caused by various species of the protozoan genus *Babesia*. Although it occurs worldwide, data relating to European infections have now been collected for many years. These data have boosted the publication record and increased our working knowledge of these protozoan parasites. Both the large and small forms of *Babesia* species (*B. canis*, *B. vogeli*, *B. gibsoni*, and *B. microti*-like isolates also referred to as "*B. vulpes*" and "*Theileria annae*") infect dogs in Europe, and their geographical distribution, transmission, clinical signs, treatment, and prognosis vary widely for each species. The goal of this review is to provide veterinary practitioners with practical guidelines for the diagnosis, treatment and prevention of babesiosis in European dogs. Our hope is that these guidelines will answer the most frequently asked questions posed by veterinary practitioners.

## Background

Towards the end of the 19th Century, Dr. Victor Babes, a Romanian physician, observed microorganisms in the erythrocytes of cattle and sheep with haemoglobinuria. These microorganisms were later named *Babesia bovis* and *Babesia ovis*, respectively, with the genus name *Babesia* after its discoverer [1]. Not long after these observations in ruminants came the first description of *Babesia* spp. infection in dogs, in Italy (1895) [2]. Currently, these protozoan diseases occur worldwide [3, 4].

Parasites of this genus are primarily transmitted through tick bites and as such can infect a wide variety of domestic and wild animals as well as humans [5]. This association arose as a byproduct of the tick’s adaptation to feed on blood. Not surprisingly, dogs are one of *Babesia* spp. many targets, with various species of *Babesia* infecting canines and causing canine babesiosis (formerly called canine piroplasmosis). Hard ticks are the main vectors for *Babesia* spp.; within the tick, *Babesia* spp. undergo the sexual conjugation and the sporogony portions of their life-cycles. These stages occur within the intestinal lumen and then within the haemocoel of the tick. A blood meal will ultimately transmit the sporozoites from the tick’s salivary gland to their new vertebrate host, whereupon the protozoan life-cycle is completed by asexual replication (merogony) within the red blood cells, where the parasites appear as merozoites.

This guide to babesiosis in dogs focuses on Europe and is aimed towards informing veterinarians working in small animal practices. This document is intended to answer the most commonly asked questions about the clinical management, including diagnosis, treatment, prognosis and prevention of these parasitic diseases, with an emphasis on the European context.

## Review

### Which species of *Babesia* can infect dogs in Europe?

Traditionally, the morphology of the protozoan (piroplasm merozoites) within the red blood cell was used as the chief taxonomic determinant. This assessment, made by microscopic evaluation of a blood smear, can be used to classify these protozoa as either large (e.g. *Babesia canis*) or small forms (e.g. *Babesia gibsoni*). Subsequently, molecular techniques allowed the identification of several species of *Babesia* that can infect dogs.

The large *Babesia* spp., previously considered to be *B. canis*, currently include *B. canis*, *Babesia rossi* and *Babesia vogeli* as distinct species [6]. Their identical morphology initially led *B. rossi* and *B. vogeli* to be thought of as subspecies of *B. canis*, although significant differences in their clinical presentation, geographical distribution and vector specificity now lead us to consider otherwise [4, 7–10]. In addition, a large-form *Babesia* species, related to *Babesia bigemina*, has been described in North Carolina in the United States [11].

Thus far, only three small *Babesia* species with clinical importance have been described: *B. gibsoni*, *Babesia conradae* [12, 13], and the recently reported "*Babesia vulpes*" [14] suggested by Baneth et al. [14] for the previously named *Babesia* "Spanish dog isolate", *Babesia* "*microti*-like", "*Babesia* (*Theileria*) *annae*", and *Babesia* cf. *microti* [15, 16], based on their natural hosts and on an apparent lack of any pre-erythrocytic stage of infection in lymphocytes. However, no types were fixed for both, "*Theileria annae*" and “*Babesia vulpes*"; therefore, these names must be considered *nomina nuda* and thus unavailable names.

In this review, we will use *Babesia microti-*like sp. to describe this infection. Interestingly, a *Theileria* spp. infection phylogenetically closely related to *Theileria* spp. found in sable has been reported in South Africa as a cause of disease with bleeding tendency associated with severe thrombocytopenia and anaemia in dogs [17, 18].

Logically, the geographical distribution of *Babesia* spp. depends on the presence of competent ticks to transmit each of them; thus far, not all such species have been identified in Europe. For the large *Babesia* species, only *B. canis* and *B. vogeli* have been found in Europe; a single record of detection of DNA of *B. rossi* needs confirmation [19]. As far as small *Babesia* species are concerned, *B. microti-*like sp. and *B. gibsoni* have been reported in several European countries [4] (Table 1).

**Table 1:** Geographical distribution, relevant vectors, and the expected size of *Babesia* spp. in blood smears. Data for the primary *Babesia* species found in Europe provided

Species	Geographical distribution	Vector	Approximate size (μm) in a blood smear	Reference
*Babesia canis*	Described across most of Europe (from Portugal to the north and east of Europe), and especially common in cool and wet climates. Higher prevalence in central Europe and lower prevalence in the Mediterranean basin	*Dermacentor reticulatus*	2.5 × 4.5	[9, 21, 55, 56, 58, 61, 84, 100, 149–151]
*Babesia vogeli*	Albania, Croatia, France, Greece, Italy, Portugal, Romania, Serbia, Slovenia, Spain and Turkey	*Rhipicephalus sanguineus*	2.5 × 4.5	[9, 21, 55, 56, 58, 61, 62, 84, 100, 149–151]
*Babesia gibsoni*	Croatia, Germany, Italy, Serbia, Slovakia, Spain and United Kingdom	*Rhipicephalus sanguineus*?^a^	1 × 3	[21, 48, 73, 152–154]
*Babesia microti*-like sp.	Croatia, France, Italy, Portugal, Serbia, Spain and Sweden	*Ixodes hexagonus* ^a^ *Ixodes canisuga* ^a^	1 × 2.5	[21, 35, 50, 62, 63]

^a^Vectorial ability has not been demonstrated in the laboratory; its role is an assumption based on epidemiological data

In addition, molecular studies reported *Theileria equi*, *Theileria annulata* and *Babesia caballi* infections detected only by polymerase chain reaction (PCR) in dogs from Spain [20], Croatia [21] and France [19]. *Theileria equi* infections have also been documented in Jordan [22], Nigeria [23] and South Africa [17]. However, the epidemiological and clinical significance of these infections in dogs remain unknown.

### What are the vectors and the geographical distributions of *Babesia* spp. causing disease in dogs in Europe?

For *B. canis*, the relevant vector is the tick *Dermacentor reticulatus*. This tick species has a relatively wide range across Europe, preferring cool and wet climates [24]. While particularly abundant in large areas of central Europe, it can be found even in isolated pockets from Portugal to Poland [25]. The association of this tick species with the transmission of *B. canis* has been documented in both field and laboratory studies [26–28], principally those conducted in France and Germany. The adult tick parasitises dogs while immature individuals feed on wild rodents and are endophilous. Adult ticks are most active during the winter months, with increased activity from October to March, if the winter is not too severe. Favourite habitats are the verges of paths that run through open fields or pastures near forests; a preference for sparse, vegetated, and sunny patches explains the tick’s affinity for paths [29].

Some experimental data have shown that the brown dog tick, *Rhipicephalus sanguineus* (*s.l.*) (hereinafter *R. sanguineus*), transmits *Babesia* species (e.g. *B. vogeli*) that infect dogs [7, 28]. *Rhipicephalus sanguineus* is abundant in Mediterranean areas, preferring temperate climates, but being endophilous can also tolerate colder regions of central Europe and the British Isles [30]. The importation of tick-infested dogs from Mediterranean regions may be a common feature for cases detected in these colder climes [30]. As yet, there are no complete data for the geographical distribution of the brown dog tick, because unfortunately, no consensus exists regarding its morphological identification [31]. The ability of this tick to survive indoors also complicates any precise determination of its restrictive range in the wild [32]. We do, however, know that hibernation (for example, in the crevices of kennel buildings) is induced as temperatures dip below 6 °C. There is also a requirement for some humidity, which can be provided artificially around buildings by ornamental water features and other artificial irrigation. Not surprisingly, mild and humid riverbanks, with their increased density of wild carnivores, are also popular areas of adult tick infestation, which peaks between May and August. The largely unnoticed larvae (hatched from tick eggs) will appear on their hosts in the summer, with the last developmental stage completing in August - September. The hibernating stage is either the engorged nymph or the newly molted adult [32]. Ticks of the complex *R. sanguineus* may serve as potential vectors for *B. gibsoni*, at least in Europe, while in Asia, its main distribution range is attributed to transmission by the tick *Haemaphysalis longicornis* [33, 34].

Details of the life-cycle of *B. microti*-like sp. are still largely unknown, but the species *Ixodes hexagonus* has been implicated as the potential tick vector, given their discovery on dogs infected with the protozoan [35, 36]. *Ixodes hexagonus* exclusively develops the "pholeophilic" (burrow-dwelling) cycle, so-called by the French researchers who reported this activity [37, 38]. *Ixodes hexagonus* is entirely absent from vegetation, and its free-living stages are confined to the den, where it commonly parasitises hedgehogs and wild carnivores such as foxes. Consequently, this tick favours areas shared with a high density of these wild carnivores and is commonly found on hunting dogs or dogs that investigate burrows, including abandoned ones. *Ixodes hexagonus*, together with a related species, *I. canisuga*, is present in practically all areas where foxes are abundant [39], including where *B. microti-*like sp. is yet to be described, which leaves some ambiguity regarding transmission. However, *B. microti-*like sp. DNA has been identified in both *I. hexagonus* and *I. ricinus* [40], as well as in *I. canisuga* [41], although no data exist to substantiate their competence as vectors for *B. microti-*like sp*.*

An official map displaying the known distribution of *D. reticulatus* can be found at http://ecdc.europa.eu/en/healthtopics/vectors/vector-maps/Pages/VBORNET-maps-tick-species.aspx. The European distribution of *R. sanguineus* has already been published [32] and updated [42]. The distribution of the other tick species acting as vectors of *Babesia* spp. to dogs is still too fragmentary to be mapped.

### Are there other modes of transmission for these infections in dogs?

Although protozoans of the genus *Babesia* undergo part of their life-cycles in the tick vector, the merozoites circulating in the blood may be transmitted to a healthy host directly by blood transfusion. This scenario has been described for *B. gibsoni* infection [43], which can also be transmitted vertically [44] and by direct contact between dogs through wounds (fighting dogs), saliva or blood ingestion [45–47]. Interestingly, most dogs reported with *B. gibsoni* infection in the United States, Australia, and Europe have been Pit Bull Terriers, a result of their fighting behaviour [45–48]. The first clinical evidence of possible vertical transmission has also been now documented for *B. canis* [49] and *B. microti-*like sp. [50].

### What are the geographical distribution and prevalence of *Babesia* spp. infections in dogs in Europe?

**Fig. 1:**
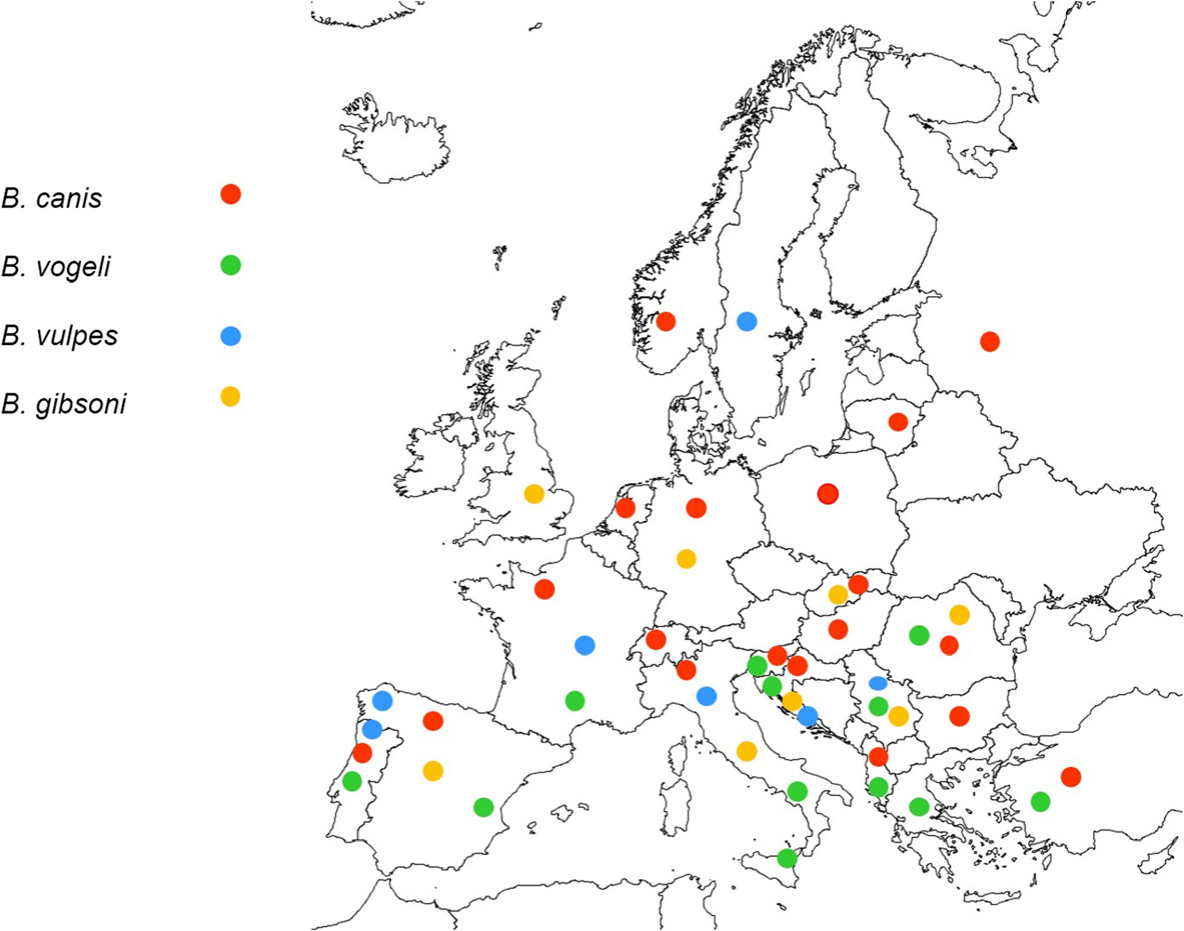
The distribution of canine *Babesia* species in Europe in dogs based mainly on molecular analysis. Note the presence of *B. canis* and *B. microti-*like sp. mostly in the cooler climate zones of north and central Europe while infection with *B. vogeli* is mainly around the Mediterranean basin. The references for each country are included in the reference list. Figure updated from Solano-Gallego & Baneth [4]

The geographical distribution of *Babesia* spp. infections in Europe is highly variable (Fig. 1) and largely dependent on the distribution of the competent tick vector. In addition, the prevalence of *Babesia* spp. infections in Europe varies (Table 2), likely because of the various diagnostic techniques used for detection, the country and population analysed, and the species of *Babesia* under investigation. Table 2 shows the prevalence of *Babesia* spp. infections in dogs across Europe. Regarding large *Babesia* species, *B. canis* has been diagnosed in various northern European countries, as well as in central and southern Europe [4, 51]. *Babesia canis* is ordinarily considered to be an infection of central Europe, principally because of the abundance of its main vector, *D. reticulatus*, in that region [52]. Results from a questionnaire-based study in France [53] indicated that the annual rate of overall incidence of *B. canis* infection seen in veterinary clinics was approximately 1 %, although some regions reported a higher incidence of up to 16 % [53]. Interestingly, three strains of *B. canis*, based on polymorphisms of the Bc28.1-gene, have been reported in Europe, with a large variation in their geographical distribution [54]. *Babesia vogeli* has been found in Turkey, Albania, Slovenia, Romania, Italy, France, Spain and Portugal [4, 55, 56]. The prevalence described for *B. canis*, using molecular methods, ranges from 2.3 % in Italy [57] to 4.6 % in Slovenia [58], 25.3 % in Poland [59], and up to 44.8 % in Romania [60]. With regard to *B. vogeli*, although the number of studies is still low, prevalence is described as ranging from 0.9 % in France [61] to 1.3 % in Slovenia [58].

**Table 2:** Prevalence of canine infection by *Babesia* spp. in Europe

Species	Country	Prevalence in % (population studied)	Technique employed	Reference
*Babesia canis*	Slovenia	4.6 (238)	PCR	[58]
	Spain^a^	1.3 (153)	PCR	[155]
	Spain^a^	10 (120)	PCR	[35]
	Italy^a^	20.7 (164)	PCR	[84]
	Italy	2.3 (420)	PCR	[57]
	Italy^b^	70 (249)	IFAT	[156]
	Poland^b^	25.3 (82)	PCR	[59]
	Croatia	2.3 (848)	PCR	[21]
	Romania	44.8 (216)	PCR	[60]
	Romania^a^	71.4 (49)	PCR	[153]
	Slovakia^c^	3.5 (366)	PCR	[157]
	Lithuania^a^	87.8 (123)	PCR	[158]
	Turkey	0.1 (757)	PCR	[159]
	France	12.9 (140)	PCR	[64]
	Bulgaria	16.2 (167)	ELISA	[160]
*Babesia vogeli*	Slovenia	1.3 (238)	PCR	[58]
	Italy^a^	6.7 (164)	PCR	[84]
	Italy^a^	4 (99)	PCR	[93]
	Croatia	0.2 (848)	PCR	[21]
	Serbia	1.9 (158)	PCR	[62]
	Spain^a^	2 (153)	PCR	[155]
	France	0.9 (108)	PCR	[61]
	France	13.6 (140)	PCR	[64]
*Babesia gibsoni* ^d^	Croatia	0.7 (848)	PCR	[21]
	Serbia	5.7 (158)	PCR	[62]
	Spain^a^	2 (153)	PCR	[155]
	Spain^a^	2.5 (120)	PCR	[35]
	Romania^a^	28.6 (49)	PCR	[153]
*Babesia microti*-like sp.	Spain^a^	1.9 (2,979)	Microscopy and PCR	[161]
	Spain^a^	62.5 (120)	Microscopy and PCR	[35]
	Spain^a^	0.7 (153)	PCR	[155]
	Croatia	0.1 (848)	PCR	[21]
	France	0.7 (140)	PCR	[64]
	Serbia	10.1 (158)	PCR	[62]

^a^Study conducted using dogs with a suspected infection, transmitted by ticks/babesiosis

^b^Study conducted using shelter dogs

^c^Study conducted in *Dirofilaria*-infected dogs

^d^Prevalence studies performed in Europe are rare, although clinical cases have been described at various locations

Concerning the small *Babesia* species, *B. microti-*like sp. isolates have been detected in dogs in Croatia [21], Serbia [62], Sweden [63], France [64], and especially in the Iberian Peninsula, specifically northern Portugal [50] and Galicia (Spain) [16, 35, 65]. *Babesia microti*-like sp. isolates have been detected in red foxes from Spain [66], Portugal [67], Italy [68], Croatia [69], Germany [41], Austria [70], Hungary [71] and Bosnia and Herzegovina [72]. Clinical cases associated with infection by *B. gibsoni* have also been described in Germany [73], Croatia [21], Italy [48], Serbia [51] and Spain [74, 75]. Unfortunately, we still lack detailed geographical distribution and prevalence data for the small-sized *Babesia* species because most descriptions are based on individual clinical case reports. However, for Croatia, molecular data have revealed a prevalence of 0.7 % for *B. gibsoni* and 0.1 % for *B. microti*-like sp. [21]. Therefore, epidemiological data of prevalence of clinical illness or subclinical infection is more limited for small *Babesia* species in Europe.

### What is the public health importance of *Babesia* infections?

None of the *Babesia* species that affect dogs and/or cats are considered to be of zoonotic importance [76]. Moreover, there is a lack of evidence that *Babesia* spp. known to be zoonotic actually have the capacity to infect dogs. However, the data for addressing this topic are incomplete given that some cases of human babesiosis are reported without any firm identification of the causative protozoan species [77].

Human babesiosis is a rare disease and primarily involves just two species of *Babesia*: *Babesia divergens*, a parasite of cattle in Europe, and *Babesia microti* that parasitises small rodents in the United States [76]. Variants of these two zoonotic species in Europe include a *B. divergens*-like species, *Babesia* sp. EU1 (also called *B. venatorum*) and a *B. microti*-like species, which is only sporadically reported [78].

Infection by *B. divergens*, following transmission by *I. ricinus*, can result in severe clinical disease, especially in immunocompromised patients (undergoing chemotherapy, splenectomised, or human immunodeficiency virus-positive). In immunocompetent individuals, it presents as a mild or subclinical infection [78].

### Is there a breed, age or sex predisposition for canine babesiosis?

A breed predisposition has been suggested in Hungary, citing the vulnerability of the German Shepherd and Komondor breeds to developing babesiosis due to *B. canis* [79]. Predisposition of other breeds for *B. canis* [80], *B. vogeli* [81], *B. gibsoni* [45] and *B. rossi* [82] infections has been described in different latitudes.

Regarding the sex preference, intact females have a lower risk of presenting babesiosis due to *B. rossi* when compared with intact or neutered males and neutered females. The reason for this predisposition has not been fully elucidated [82], but it has been suggested that testosterone causes prolonged and more intense *B. microti* parasitemia in infected male rodents [83].

Young dogs are more likely to present severe babesiosis when infected by *B. canis*, *B. vogeli* [4, 84] or *B. rossi* [85]. Similarly, old hunting dogs infected by *B. microti*-like sp. are reported to have a greater risk for developing azotemia [86].

### What clinical signs and laboratory abnormalities are found in dogs infected with *Babesia* spp.?

The clinical manifestations found during the course of *Babesia* spp. infections vary, ranging from subclinical infections to multi-organ failure, with a risk of death [87]. While the spectrum of disease may appear daunting, the collection of an extensive history and clinical presentation data backed by laboratory abnormalities should allow the veterinarian to shorten the list of differential diagnoses [4]. The history and common and diverse clinical signs and laboratory abnormalities observed among the *Babesia* species, the course of several types of *Babesia* infection, and prognoses are shown in Table 3.

**Table 3:** Primary clinical manifestations and prognosis for dogs infected with the different species of *Babesia* found in Europe [4]

	*B. canis*	*B. vogeli*	*B. gibsoni*	*B. microti-*like sp.
History and features	Young dogs, adult dogs, hunting dogs/sheepdogs (German Shepherd and Komondor) that live outdoors. A greater number of cases is seen in autumn, and spring	Puppies or adult/older dogs with concomitant infectious or non-infectious diseases	Common in fighting dogs (Pit Bull Terrier and Tosa)	Young, adult dogs, guard/hunting dogs that live outdoors
Severity of disease	Moderate to severe	Mild to moderate	Moderate to severe	Moderate to severe
Clinical signs and laboratory abnormalities that differ among *Babesia* spp.	Petechiae, epistaxis, vomiting, lymphadenomegaly, hypotension, low T3 syndrome, mild to moderate nonregenerative, normochromic, and normocytic anaemia, regenerative anaemia (less common), leukopenia with neutropenia and/or lymphopenia, hypoalbuminemia, elevation of liver enzymes (ALT, AST, ALP), hypokalemia, hyponatremia, and hyperchloremia, hyperlactatemia, hyperphosphatemia, hypertriglyceridemia, hypoglycemia, prerenal and renal azotemia	Regenerative immune-mediated hemolytic anaemia, nonregenerative anaemia, leukocytosis and leukopenia	Lymph node enlargement, enlargement of the spleen, small-bowel diarrhea, weight loss, protein-losing nephropathy, PU/PD, and abdominal effusion. Mild to severe regenerative immune-mediated hemolytic anaemia, neutropenia and leukocytosis. Hypoalbuminemia, azotemia and elevation of liver enzymes (ALT, ALP)	Azotemia, proteinuria, cylindruria and hyperglobulinemia
Course of infection related to disease manifestation	Acute	Acute and chronic	Acute and chronic	Acute and chronic?
Prognosis	Good to poor	Good	Guarded to poor	Guarded to poor
Reference	[84, 89, 106, 131, 162–167]	[84, 106, 162]	[48, 102, 132, 168–171]	[16, 35, 86, 172]

Common clinical signs and laboratory abnormalities among *Babesia* spp.: Fever, lethargy, anorexia, pale mucous membranes, weakness, bounding pulse, jaundice, pigmenturia, mild to severe thrombocytopenia, mild to severe regenerative anaemia due to hemolysis, bilirubinemia, bilirubinuria, and haemoglobinuria

The wide range of clinical manifestations depends very much on the species of *Babesia* causing infection and other factors that affect the severity of the disease, including age, splenectomy, immune competence, and concomitant infection or disease [4, 10]. In addition, disease severity has been associated with parasite density in *B. rossi* infection [88]. However, limited information is available regarding disease severity and parasite density in other *Babesia* species. In a recent study, parasite density was not different between survivors and non-survivors in dogs infected with *B. canis* [89]. It is likely that different *Babesia* spp. might result in different parasitemias due to differences in disease severity, but further studies need to confirm this hypothesis.

Differences in virulence have been described among *Babesia* species infecting dogs. In general, it is assumed that the least pathogenic large-sized species of *Babesia* is *B. vogeli*, at least for adult dogs, and that the most virulent species is *B. rossi*, which is probably found only in Africa. The pathogenicity of small-sized *Babesia* spp., such as *B. gibsoni* and *B. microti*-like sp., is moderate to severe [4, 10, 90].

There are clinical signs and clinicopathological abnormalities that are common across all *Babesia* species infecting dogs (Table 3). Frequent clinical signs associated with canine babesiosis are apathy, weakness, anorexia, pale mucous membranes and a poor general condition. All *Babesia* species can cause fever, enlarged lymph nodes and spleen, anaemia, thrombocytopenia, jaundice and pigmenturia. Although thrombocytopenia, to a varying extent, is frequently detected, the presence of petechiae or ecchymosis is less common. Thrombocytopenia, when present, varies from mild to severe, as does anaemia. Other abnormalities that can be detected include hypoalbuminemia and hyperbilirubinemia [4, 10]. Depending on the infective species and the course of infection, anaemia can be regenerative; nonregenerative anaemia is more typically associated with *B. canis* [84]*.* In all species, anaemia is caused by a combination of intravascular and extravascular hemolysis resulting from parasite-caused injury and rupture of red blood cells, the cells’ increased osmotic fragility, and the activity of secondary immune-mediated processes.

Some clinical signs and clinicopathological abnormalities differ among *Babesia* species infecting dogs (Table 3). Many dogs could present other clinical signs that are not directly related to hemolysis by piroplasms but that demonstrate the involvement of other organs. These complications are especially prevalent following infection by *B. rossi*. A non-exhaustive list includes weight loss, acute or chronic nephropathy, glomerulonephritis, coagulation disorders (disseminated intravascular coagulation), jaundice from liver disease, immune-mediated hemolysis or thrombocytopenia, hemoconcentration, shock, metabolic and/or respiratory alkalosis, and/or acidosis, gastrointestinal disorders (vomiting or diarrhea), pancreatitis, ascites, ocular lesions (uveitis or blindness), myalgia, rhabdomyolysis and respiratory problems (edema or acute respiratory distress) [91].

It must be noted that many “carrier” dogs with chronic infections will not present with any clinical signs as the result of premunition or concomitant immunity unless their health deteriorates, for example from immunosuppressive treatment, splenectomy, or any other immune-compromised situation (e.g. post-surgical stress or debilitating disease). This phenomenon has been described in Greyhounds infected by *B. vogeli* and in Pit Bull Terriers infected by *B. gibsoni* [45, 92]. It results from the inability of the immune system to eliminate the infection, which then establishes itself with more rigour when the immune system is in abatement [4, 10].

### Is light microscopy evaluation of a blood smear useful for the diagnosis of canine babesiosis?

**Fig. 2:**
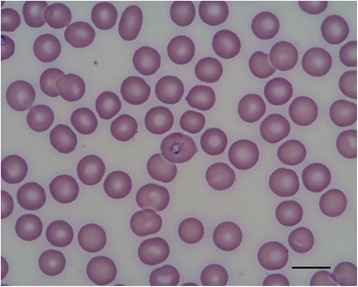
Photomicrograph showing a large-sized *Babesia* spp. (*B. canis*) in canine erythrocytes. *Scale-bar*: 10 μm

**Fig. 3:**
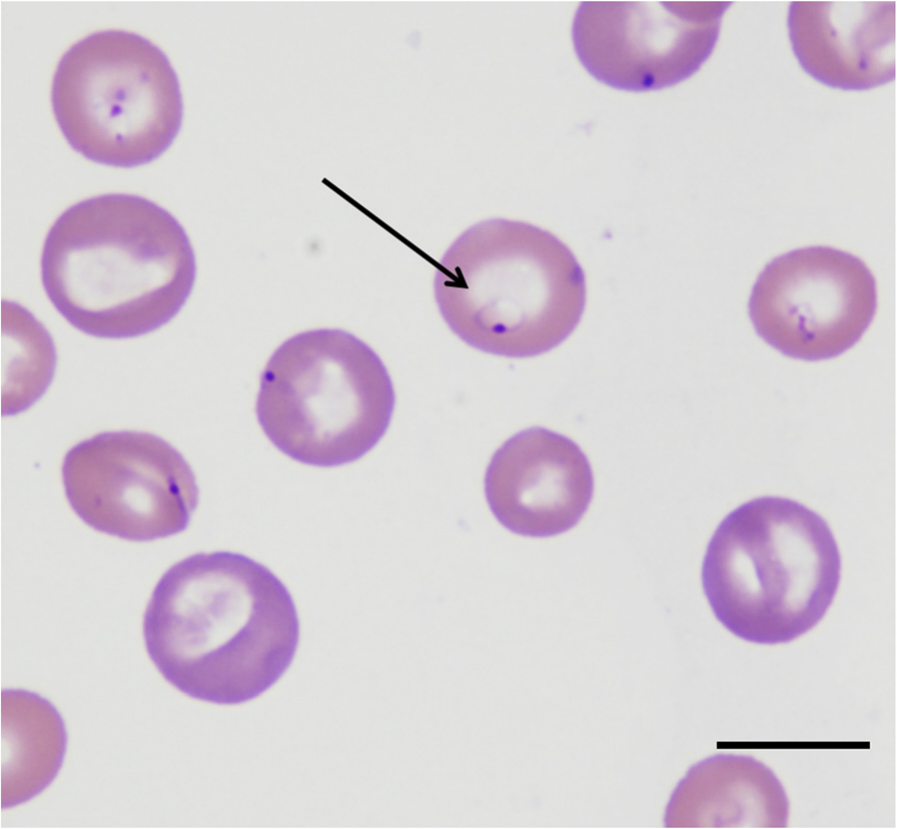
Photomicrograph of a small-sized *Babesia* spp. (*B. gibsoni*, *arrow*) in canine erythrocytes. *Scale-bar*: 10 μm

**Fig. 4:**
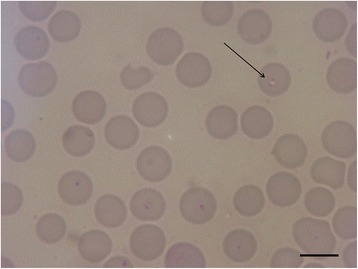
Photomicrograph of a small *Babesia* (*B. microti*-like sp., *arrow*) in canine erythrocytes. *Scale-bar*: 10 μm

Blood smear examination is a useful diagnostic tool for clinical babesiosis in dogs. Microscopy evaluation continues to be the easiest and most accessible diagnostic test for most veterinarians. However, the sensitivity of this method is lower than that of molecular diagnosis in assisting the veterinarian in making a positive diagnosis and is rather dependent on the species infecting the dog. The two forms of *Babesia*, large (Fig. 2) and small (Figs. 3, 4), can be distinguished using a blood smear. Although light microscopy is highly specific and can be used to diagnose the majority of sick dogs infected by the large forms of *Babesia* (e.g. *B. canis*) [4, 84], it is less commonly detected in *B. vogeli* infections [84]. For this infection, more sensitive molecular PCR-based methods, are more appropriate [93]. The small piroplasms (*B. gibsoni*, *B. microti*-like sp.) are hard to observe by light microscopy, which has relatively poor to moderate sensitivity [35], and expertise is needed.

**Fig. 5:**
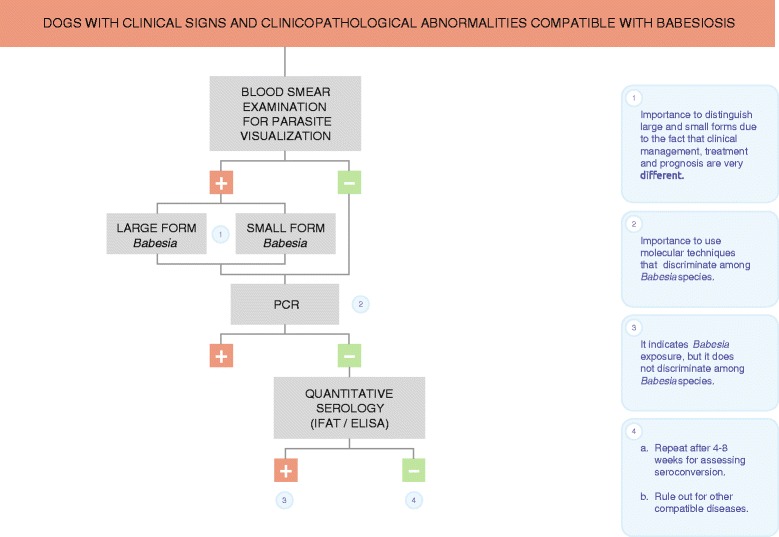
Diagnostic algorithm for canine babesiosis

Blood smear observation should therefore be a “first step” diagnostic tool, with negative blood smears reassessed by PCR using blood or splenic tissue (Fig. 5). In addition, to identify the species of piroplasm, morphological observation is insufficient, and molecular techniques such as PCR and sequencing are necessary. Fresh blood is always recommended for the smear. Additionally, observation of large *Babesia* spp. in a capillary blood smear, such as that obtained from the ear or nail, seems to be more easily accomplished thanks to the greater abundance of the parasite in this type of sample [10, 88]. The limit of detection of parasites in a thin blood smear appear to be parasitemias of 0.5 % [88].

### What serological techniques can be used to diagnose babesiosis?

The serological tests that can be used are quantitative techniques, such as indirect immunofluorescence (IFAT), or enzyme-linked immunosorbent assay (ELISA). One of the advantages of IFAT or ELISA is that these tests allow us to determine the antibody levels and therefore establish whether they are high or low. For this reason, it is important to send the samples to a laboratory that routinely uses quantitative serological techniques and can provide a final titre by IFAT or optical density by ELISA [42]. Rapid techniques are not yet commercially available for the detection of anti-*Babesia* antibodies for the clinical setting and will offer only a “positive/negative” result, without providing an antibody titre or level. Furthermore, quantitative techniques are generally more sensitive and specific than rapid techniques. Currently, no universal antigen has been developed for screening using routine diagnostic serology against all *Babesia* species that infect dogs. The most commonly used antigen in practice and research is that for *B. canis*; *B. gibsoni* [94, 95] and *B. microti*-like sp. [35] antigens are also available, but information is scarce regarding these antigens. In addition, the specificity and sensitivity of these techniques are not well established [4]. Therefore, the scope for diagnosis by serology is extremely limited and requires further investigation. Nevertheless, false-negative PCR results have been reported in chronic babesiosis involving *B. gibsoni*, attributed to parasite elimination from the circulating blood by the host. These data show that in the long term (up to 420 days post-infection), an infection might be revealed by serology only retrospectively [95].

### How should we interpret a positive serological result for *Babesia* spp.? Do serological cross-reactions exist among different species of *Babesia*?

The interpretation of a positive result for *Babesia* when using a serological technique is complicated by cross-reactivity among the different species. In general terms, there is significant cross-reactivity between different species of *Babesia*, especially the more phylogenetically related species. For example, cross-reactivity for large *Babesia* spp. (*B. canis* and *B. vogeli*) can result in matching antibody levels across species. Cross-reactions can also exist between small *Babesia* spp. (*B. gibsoni*) and large species (*B. canis*) [96] as well as between small *Babesia* spp. (*B. gibsoni* and *B. microti*-like sp.) or between large *Babesia* spp. (*B. canis*, *B. vogeli* and *B. rossi*) [28].

For this reason, a positive result would indicate exposure to infection by *Babesia* but not precisely identify which species. A positive serological result can also indicate a past or current infection. Despite their infrequent use in the clinical setting, molecular techniques would provide a more informative diagnosis.

### Could a seronegative dog be infected with *Babesia* spp.?

It is perfectly feasible for a dog to be seronegative and infected with *Babesia* because infections by species such as *B. canis* manifest acutely [4, 97]. Consequently, the three to four-week lag in post-infection antibody production would provide a serologically negative window. Therefore, seroconversion could be used as a serological technique to confirm acute infection by *Babesia* spp. In these cases, initial quantitative serology should be performed when the patient first presents with clinical signs and/or laboratory abnormalities. Subsequently, quantitative serology should be performed again after 4–8 weeks (see Fig. 5). Medium to high positive antibody levels during the convalescent phase (at least 3–4 weeks after infection) can confirm infection by *Babesia* at the time of presentation [35]. However, data are limited about the usefulness of seroconversion in canine *Babesia* infections. Seroconversion is not commonly employed in clinical practice.

### Why is the amplification of DNA by PCR useful in the diagnosis of babesiosis? What biological samples should be chosen to perform a molecular diagnosis of *Babesia* spp. infections?

In general terms, PCR is very useful in diagnosing babesiosis. First, PCR detection is more sensitive than a direct blood smear examination. Secondly, the detection of DNA for a specific pathogen in a clinical setting can be considered evidence of an active - and therefore ongoing - infection. In addition, unlike direct detection by light microscopy or serology, PCR allows a more reliable identification of the causative species infecting the dog [4].

Different molecular techniques allow the identification and differentiation of the various species of *Babesia.* These include semi-nested PCR [98], reverse line blotting [99, 100], and PCR-restriction fragment length polymorphism analysis [101]. In addition, several genes are commonly used to discriminate among *Babesia* species. Typically, these include the nuclear ribosomal RNA genes [7, 8] and the two internal transcribed spacers (ITS1 and ITS2) [7]. These molecular techniques allow us to refine our diagnosis to the species level and thus provide a more accurate prognosis. Finally, PCR DNA amplification can be a useful technique for monitoring treatment [102].

Ideally, peripheral blood buffered with ethylenediaminetetraacetic acid (EDTA) should be used to conduct molecular analyses using PCR. Moreover, splenic tissue can also be useful, although, as mentioned below, this sample is not usually pursued because it involves a more invasive procedure [4].

### Are co-infections common in dogs infected with *Babesia* spp.? What is the clinical importance of a co-infection for the progression of disease?

Co-infections with *Babesia* spp. are not well documented and are rarely reported in dogs. However, sequencing and phylogenetic analyses suggest that the diversity of piroplasm species that co-infect dogs may be greater than previously thought [15, 21]. A study conducted with 120 Spanish dogs from Galicia and Asturias, all with clinical signs compatible with babesiosis, demonstrated the presence of *B. microti*-like sp. in 75 dogs (62.5 %), with 15 dogs positive for other piroplasmid species (12 for *B. canis* and three for *B. gibsoni*) [35]. Co-infection with other agents was not detected, possibly indicating that co-infection with other pathogens is not common [35]. Moreover, an interesting study found a high percentage of co-infection with different strains of *B. canis* in dogs from France [39]. In addition, co-infection with *B. rossi* and *B. vogeli* and a triple infection with *B. rossi*, *B. vogeli* and *Ehrlichia canis* have been reported in South African dogs [103]. In contrast to the rare occurrence of co-infection with different species of *Babesia*, it could be common in endemic areas to find dogs co-infected with other pathogens such as *Leishmania* spp.*, Ehrlichia*/*Anaplasma* spp., *Hepatozoon* spp. or *Rickettsia conorii*, depending on the geographical area and the distribution of the competent arthropod vectors [93, 104]. Additionally, it should be noted that co-infection is of major clinical importance for several reasons: it complicates diagnoses, exacerbates clinical signs, reduces effectiveness of treatment, and can worsen the prognosis [104].

### What is the treatment of choice for *Babesia* infections?

Despite the large number of clinical cases and uncontrolled experimental studies, little robust scientific evidence is available regarding the treatment of canine babesiosis; Table 4 displays those currently used in dogs. Imidocarb dipropionate is the treatment of choice for canine babesiosis caused by the large *Babesia* species. One dose of 6.6 mg/kg intramuscularly (IM) or subcutaneously (SC) is the recommended treatment. Although some authors suggest an additional dose of imidocarb (separated by 15 days for *B. canis* and *B. vogeli* infections), if the dog does not respond adequately, it may be wiser to reconsider the diagnosis. Moreover, this approach is not the treatment of choice for small *Babesia* species (*B. gibsoni* and *B. microti*-like sp.). The most frequently described side effects associated with this drug are pain at the injection site and cholinergic signs (anorexia, hypersalivation, epiphora, abdominal pain, vomiting and diarrhea), which generally disappear quite quickly, although these latter effects can be ameliorated by pre-medicating with atropine or glycopyrrolate [12, 16, 105–107]. The toxic effect of an overdose of imidocarb dipriopionate is nephrotoxicity.

**Table 4:** Treatments used for infections with the various species of *Babesia*

Species	Drugs	Efficacy	Dosage	References
*Babesia canis*; *Babesia vogeli*	Imidocarb dipropionate	Good	6.6 mg/kg IM/SC once (can be repeated after 15 days)	[105, 106]
	Doxycycline	Poor	10 mg/kg/day PO, 30 days	[117]
*Babesia microti*-like sp.	Imidocarb dipropionate	Poor	6.6 mg/kg IM/SC once (can be repeated after 15 days)	[12, 16, 105, 106]
	Azithromycin + Atovaquone	Good to moderate	10 mg/kg PO SID/10 d + 13.5 mg/kg PO TID/10 days	[109, 113]
	Azithromycin + Buparvaquone	Good to moderate	10 mg/kg PO SID +5 mg/kg IM (repeat after 48 h)	[113]

The combination of atovaquone and azithromycin is the only treatment that has been proven to reduce parasitemia with *B. gibsoni* below the PCR limit of detection. Atovaquone is an anti-parasitic drug that inhibits the action of cytochrome *b*. The most commonly used dose of atovaquone is 13.5 mg/kg, administered *per os* (PO) every 8 h with fatty food (to maximise drug absorption) and in combination with azithromycin (at a dose of 10 mg/kg PO) for ten days. This drug combination also seems to be effective in treating infections with other small *Babesia* species like *B. conradae* [108] and is likely to be useful in treating *B. microti*-like sp. infections. Nonetheless, it seems that in some cases, a lower rate of success with atovaquone treatment is being reported [102, 108–112]. Recently, the use of two IM doses of buparvaquone at 5 mg/kg separated by 48 h has demonstrated good clinical efficacy in dogs naturally infected by *B. microti*-like sp. in Spain, with results superior to those achieved with atovaquone [113].

Diminazene also seems effective against *B. canis* when administered IM as a single dose of 3.5 mg/kg. However, it does not have the same efficacy against *B. gibsoni*, although it does reduce parasitemia, morbidity and mortality. Side effects include neurological abnormalities, which can be severe on overdose. Its use is currently restricted to clinical cases that are refractory to other treatments [114, 115], and it is not commonly used in Europe. The use of combined clindamycin, diminazene and imidocarb dipropionate may also be promising in the treatment of *B. gibsoni*, as compared to the combination of atovaquone and azithromycin [116].

Antibiotics are not the treatment of choice for piroplasmosis. Nonetheless, doxycycline has been described as lessening the severity of clinical signs and is associated with a reduction in morbidity and mortality for *B. canis* and *B. gibsoni* infections [117, 118]. The most commonly used dose is 10 mg/kg/day, administered PO or (sporadically) intravenous (IV). In case of vomiting, the recommendation is to split the dose into 5 mg/kg given every 12 h [117]. Clindamycin has been used in the treatment of *B. gibsoni* infection at a dose of 25 mg/kg, administered PO every 12 h for 14 days and has been shown to reduce clinical signs and laboratory abnormalities [119]. It is important to remember that antibiotics alone will not eliminate the infection. However, combinations of different antibiotics have some efficacy in treating dogs infected with *B. gibsoni*. Examples include the combination of clindamycin (11 mg/kg every 12 h PO), metronidazole (15 mg/kg every 12 h PO), and doxycycline (5 mg/kg every 12 h PO); or enrofloxacin (2.5 mg/kg every 12 h PO), metronidazole (5–15 mg/kg every 12 h PO), and doxycycline (7–10 mg/kg every 12 h PO) [118, 120]. In summary, because of the scarce scientific evidence regarding the efficacy of antibiotics in treating canine babesiosis, their use in these diseases should be restricted.

Other treatments used with varying success to treat babesiosis in dogs include quinuronium sulfate, trypan blue solution and pentamidine; experimental treatments include artesunate, plant extracts or tick peptides [114, 121–125].

### Are there other supportive therapies that could be used for babesiosis?

Supportive treatment is provided only to dogs admitted for inpatient hospital-based care. Supportive care is required for moderate to severe babesiosis. It is difficult to characterise the proportion of cases that need supportive treatment, which varies depending on the type of *Babesia* species infecting the dog.

In dehydrated or hypovolemic dogs, the use of intravenous crystalloid fluid therapy is indicated, together with the correction of electrolyte and acid–base abnormalities. Fluid therapy is also essential for maintenance of blood volume and adequate end-organ perfusion, diuresis and prevention of red blood cell sludging in capillaries [10, 126]. In dogs with clinical signs associated with anaemia, packed red blood cell transfusions should be provided using pre-screened units; alternatively, synthetic haemoglobin can be used. Dogs with disseminated intravascular coagulation or coagulation disorders may require plasma transfusions.

The use of immunosuppressant drugs in dogs with immune-mediated haemolytic anaemia (IMHA) or thrombocytopenia is controversial because these conditions are always associated with infectious disease. If the dog is stable and does not require hospitalisation, treatment should be restricted to antiprotozoal agents; treatment should not be initiated exclusively in relation to the hematocrit or platelet value but rather based on the clinical signs associated with the anaemia or thrombocytopenia. Thus, occasionally, dogs with a hematocrit or platelet value of less than 15 % or 40,000/μl, respectively, may manifest good progress when treated with anti-babesial therapy alone. If, despite antiprotozoal treatment, the dog has moderate-to-severe clinical signs (or a high risk for them), such as sudden collapse or spontaneous bleeding associated with IMHA (e.g. severe spherocytosis, autoagglutination, anti-erythrocyte antibodies or positive Coomb’s or antinuclear antibody tests) and/or immune-mediated thrombocytopenia (when platelets/μl are between 20,000 and 40,000), the use of 2 mg/kg/day of prednisone is recommended because prognosis is guarded to poor with reported mortality rates of 28–70 % [127, 128]. However, because no laboratory test or hematological parameter allows the clinician to decide if the immunosuppressant treatment is really necessary, in the majority of such cases, a short course of treatment (ten days or less) is sufficient for secondary IMHA or thrombocytopenia. Moreover, a dose reduction could be implemented more rapidly than usual when there are primary immune-mediated alterations. Other immunosuppressant drugs have not shown the same efficacy and are therefore not recommended. Dogs previously treated with immunosuppressant drugs over a sustained period of time before their treatment for babesiosis do not have as good a clinical response and may be predisposed to other infections and/or relapses [4, 10, 126]. Pulmonary thromboembolism is a common cause of death in dogs with IMHA. Therefore, heparin, acetylsalicylic acid or clopridogrel might be used as a thromboprophylaxis in dogs with IMHA [129].

Many other supportive therapies may be beneficial depending on the clinical signs and/or laboratory abnormalities, both those caused by the babesiosis directly and those resulting from its treatment with antiprotozoal agents. For example, anti-emetics should be used to counter vomiting, or oxygen therapy should be used when there is respiratory distress [126, 130].

### What is the expected clinical response following the treatment of babesiosis?

The majority of dogs infected with large piroplasms (*B. canis* and *B. vogeli*) improve clinically in days 1–7 after specific antiprotozoal treatment, although some dogs will not respond until more than 15 days have passed. Dogs infected with *B. canis* or *B. vogeli* will generally manifest a complete recovery following their treatment [90, 131]. In general, the clinical response is good and more rapid (24–48 h) in dogs infected with the large *Babesia* species than with small [4, 10]. However, a recent study reported a high mortality rate (53 %, 8 out of 15) in dogs with babesiosis due to *B. canis* during the first 24–48 h after clinical presentation and treatment [89]. In canine *B. canis* infections, poor outcome and mortality are associated with moderate anaemia, severe thrombocytopenia, mild to moderate leukopenia, hyperlactatemia, moderately increased serum phosphate and triglyceride concentrations and moderately decreased total serum protein concentrations [89].

Some dogs infected with *B. gibsoni* and treated with atovaquone and azithromycin do not show relapse of the disease, and some remain PCR-negative for several years. However, dogs that remain infected with *B. gibsoni* following treatment may present with a different clinical picture. They may demonstrate a complete resolution of anaemia, without clinical recurrence after stress (including unrelated, disease-mediated), although occasionally, mild thrombocytopenia or hypergammaglobulinemia persists. For some dogs, clinical signs may disappear entirely, but moderate anaemia, thrombocytopenia or hypergammaglobulinemia will persist. Finally, in some other dogs, the clinicopathological abnormalities may be resolved but can reappear under stressful circumstances, which is especially common in splenectomised dogs [102, 132].

In general, little information is available regarding the clinical response of dogs infected with *B. microti*-like sp. isolates. We would predict that their clinical progression would be similar to that described for dogs infected with *B. gibsoni*, likely because of the lack of any truly effective treatment [16]. However, azotemia has been reported to be the main cause of death for *B. microti*-like sp.-infected dogs, with a mortality rate of 22 % [86], although a pre-renal azotemia was not fully ruled out. Furthermore, a recent study carried out in the same area did not yield the same findings [35]. In general, these dogs have a very poor response to protocols using imidocarb dipropionate [113]. Other therapeutic alternatives are being considered, such as combinations of atovaquone or buparvaquone with azithromycin. In the majority of dogs treated with these combinations, the trend is towards a favourable initial clinical response [113], but further follow-up studies are needed to evaluate and compare relapse intervals for the various protocols. Therefore, follow-up blood tests (complete blood count and biochemical profile) are needed until hematocrit, platelet concentration, and liver/kidney abnormalities normalise. This is especially important in splenectomised dogs or those dogs infected with *B. gibsoni* or *B. microti*-like sp.

### How do antibody levels evolve following treatment?

In general, antibody levels to *Babesia* spp. start decreasing three weeks after initiating treatment and decrease gradually thereafter over approximately 160 days [133]. Antibody results should always be interpreted with caution because elevated antibody levels have been reported to routinely correlate with persistent infection [134]. For some species of *Babesia* (e.g. *B. gibsoni*), it is normal to find positive antibody levels following treatment, which complicates the interpretation of positive results [111]. Taken together, these limitations, along with those already described for serological analyses, make it inadvisable to employ these techniques in disease follow-up.

### Why is PCR useful after treatment?

PCR is a useful screening strategy given that many dogs remain chronically infected with piroplasms. Their chronically infected status predisposes these dogs to relapse or to the maintenance of a chronically abnormal - and therefore injurious - clinical state. Under these circumstances, PCR can be used to establish whether the infection remains or has been most likely cleared [4, 10].

PCR should be performed before interrupting treatment and approximately 2 months after the completion of treatment, especially when monitoring small *Babesia* species. Additionally, because the sensitivity of PCR for piroplasms in whole blood is less than 100 %, it may be advisable to perform two consecutive PCR tests, separated by at least 15 days [102, 111]. In addition, true parasitic clearance can be rigorously demonstrated only if PCR is performed using multiple tissue aspirates, such as from the spleen, not just peripheral blood [10]. The prohibitive cost of several splenic PCR tests tends to restrict its use to the research setting and to proof-of-principle studies for therapeutic response.

### Can canine babesiosis be cured?

Clinical cure and a good therapeutic response are much more likely achieved for infections by large-sized *Babesia* species than infections by the small-sized species, the latter of which tend to be more refractory to conventional treatments [4]. Several therapeutic protocols aimed at infections caused by small *Babesia* species are used, although parasitological cures are considered rare. The persistence of *B. gibsoni* in dogs following treatment with different protocols using clindamycin, metronidazole, doxycycline, diminazene, imidocarb dipropionate, atovaquone, and azithromycin is testament to the resilience of this parasite [111, 112, 116, 119].

### Can dogs be re-infected by *Babesia* spp.?

The same dog can be re-infected by identical *Babesia* species or co-incidentally with a second species. Although the clinical consequences of re-infections are not well defined, in endemic regions, it is possible for dogs to be chronically infected, in a premunition phase, without clinical consequences; this phase may even be beneficial in terms of protecting against future infection [133].

### What tick control measures can be implemented to prevent infection by *Babesia* spp. in dogs?

The predominant emphasis for the prevention of babesiosis in dogs has been to focus on tick control. However, this approach is complicated by the endophilic nature of at least some of the ticks involved in its transmission. The efficient transovarial transmission of *Babesia* species in the tick implies that tick populations in endemic areas can remain infected for a long time and that dogs in contact with tick-infested areas will routinely become re-infested and exponentially amplify the tick population.

Tick prophylaxis should cover the entire period during which ticks are active, depending on the level of risk and lifestyle of the dogs. This prophylaxis may consist of regular checking of the pet for ticks by the owner and veterinarians and the regular use of acaricidal treatment.

Actions for the prevention of transmission of *Babesia* infections should focus first and foremost on the following:

1. Any attached tick should be removed. Pet owners should be aware of the importance of removing ticks as soon as possible. A large variety of purpose-designed tick removal tools are available (these may be used for removal of ticks attached to the skin; oil, alcohol or ether are not recommended).
2. Dogs and cats travelling to regions with ticks and endemic for babesiosis should also receive a regular acaricidal treatment, particularly if this disease is not endemic in their area.
3. Use acaricides with a residual action and water resistance.
4. Engaging in tick control, applying a good knowledge of tick seasonality. Ticks may be active and parasitise dogs above an ambient average air temperature of 12 °C. Below this temperature, it becomes difficult for dogs to become infested, which makes tick control much easier. Although tick control is classically recommended between spring and autumn, recent studies suggest that in some areas, it should be applied all year around [135, 136]; however, this recommendation should be considered according to local conditions.
5. The removal of ticks from kennels is unfeasible; therefore, the only and safe way to avoid the colonisation of kennels and premises by ticks is the protection of dogs, which are the only “carriers” of ticks.

Principle active compounds considered to be effective for the treatment and prevention of tick infestations in dogs include a wide variety of ectoparasiticides, which have shown different effects on ticks (repellency, antifeeding effect, disruption of attachment, expellency and/or killing effect), so it is important to use suitable acaricides to kill the ticks as quickly as possible before pathogens are released. Considering babesiosis, it is even better to prevent ticks from attaching (tick repellency *sensu stricto*) [137].

A broad spectrum of acaricidal products are licensed for use in dogs all over Europe. The different presentations include long-lasting efficacy collars (6–8 months), spot-on pipettes (3–5 weeks), sprays (2–3 days) and the new oral chewable tablets (1–3 months). These new oral molecules are systemic acaricides; thus, ticks have to attach to the host and start to feed to encounter the active ingredients.

In addition, reports are still rare regarding the efficacy of acaricidal products to prevent *Babesia* infection. These studies are mainly confined to *B. canis* transmission by *D. reticulatus* in dogs with percentages of protection that range between 88 and 100 % with a duration from 4 weeks to 3 months (Table 5).

**Table 5:** Acaricides with proven preventive efficacy against *Babesia* spp*.* transmission by *D. reticulatus* and *R. sanguineus* ticks in dogs

Principle active (Brand name, presentation - company)	Percentage of protection of *B. canis* infection (time period of evaluation)	Acaricide efficacy against *D. reticulatus* in % (time period of evaluation)	Reference
Afoxolaner (NexGard®, chewable tablets - MERIAL)	100 (56 days)	99 (4 weeks)	[173]
Fipronil + Permethrin (Frontline Tri-Act®, Spot on - MERIAL)	94.3 (4 weeks)	98.3 (28 days)	[174]
Fluralaner (Bravecto®, chewable tablets - MSD)	100 (12 weeks)	99.2 (86 days)	[175]
Flumethrin + Imidacloprid (Seresto® Collar - BAYER)	100 (4 weeks)	96.0 (48 h), 100 (4 days)	[176]
	Percentage of protection of *B. vogeli* infection (time period of evaluation)	Acaricide efficacy against *R. sanguineus* in % (time period of evaluation)	
Flumethrin + Imidacloprid (Seresto® Collar - BAYER)	100 (1 year)	99.7 (48 h)	[177]
Imidacloprid + Permethrin (Advantix®, spot on - BAYER)	88.3–94.4 % (12 months)^a^	95–100	[178, 179]

^a^The molecular diagnosis was *Babesia* spp. Identification to the species level was not carried out. However, the studies were performed in the south of Italy, so that the species of *Babesia* infecting dogs was most likely *B. vogeli*

According to the registration of these compounds in Europe, all of them can kill the already feeding tick before the estimated 48 h of sporogony of *Babesia* spp. in ticks [138], necessary for its transmission to dogs, which starts when the tick begins to feed. Of note, dogs exposed experimentally to *D. reticulatus* containing *B. canis* tested positive for *Babesia* (PCR, blood smears) after a 72-h infestation [139].

Different reports have documented the efficacy and speed of killing ticks under natural infestations or after exposure to experimental infestations when dogs are treated with the different ectoparasiticides available. In general, they are all useful, and the veterinarian should tailor the best choice considering each individual dog (lifestyle, outdoor activities, working dogs, human–animal bond).

ESCCAP (European Scientific Counsel Companion Animal Parasites) have therapy tables in each country with the whole portfolio available in Europe (www.esccap.org).

### Are there vaccines available to prevent babesiosis in dogs?

A few commercially available vaccine options exist to prevent *Babesia* infection in different animal species. For the European market, a vaccine to protect dogs against *B. canis* is available, called Pirodog® (Merial). This vaccine, which comprises soluble parasite antigens obtained from culture media supernatant [140–144], induces a partial protection for dogs newly exposed to *B. canis*, which both shortens and diminishes the severity of their clinical signs. Vaccination does not prevent infection but appears to block initiation of many of the pathological processes involved in the disease; moreover, a lower parasitemia may result. This vaccine can be administered from 5 months of age and requires annual re-vaccination but does not cross-protect against other *Babesia* species. Vaccines against other *Babesia* species such as *B. gibsoni* are currently being developed [145, 146].

### Are there other options available to prevent canine babesiosis?

Although it is fair to say that the best way to control babesiosis is to counter the vector itself, alternatives other than vaccine development have been studied. One possibility is a chemoprophylactic approach with a few dated reports showing the effectiveness of the carbanilide derivative imidocarb dipropionate against *B. canis* infection*.* In one instance, 6 mg/kg administered in three doses, each separated by one week, resulted in no improvement [105]. However, a second study using a single subcutaneous dose (again, 6 mg/kg) demonstrated protection for two weeks [147]. Doxycycline, used at a dose of 5 mg/kg/day, reduces the severity of disease in dogs infected experimentally with an extremely pathogenic isolate of *B. canis* [117]. However, the chemoprophylactic use of these drugs should be restricted to immunosuppressed dogs (primarily splenectomised dogs) with an increased risk of exposure, including their residence in endemic areas. For these patients, strict clinical and parasitological follow-ups are needed using serological diagnoses and supported with daily blood smears for at least two weeks post-surgery [10]. The demonstration of *Babesia* spp. transmission after blood transfusion, even when using donor dogs that appear to be clinically healthy [43], reinforces the need to test all prospective canine blood donors with serology and PCR assays before transfusion [148]. Dogs positive by either or both methods should not be used for blood donation.

## Conclusions

Information on canine babesiosis in Europe has significantly increased in the last few years. The present guidelines aim to answer common questions about the aetiology, epidemiology and transmission routes, clinical signs, laboratory findings, diagnosis, treatment and prophylaxis of infections caused by *Babesia* spp. We also hope that this review will contribute to the understanding of the current status of these diseases in Europe. It is important to highlight that canine babesiosis represents a group of diseases and that many species can infect dogs in Europe. Therefore, accurate detection and species recognition is crucial for selecting the most appropriate treatment and determining the most accurate prognosis.

## Abbreviations

ALP, Alkaline phosphatase; ALT, Alanine aminotransferase; AST, Aspartate transaminase; ELISA, enzyme-linked immunosorbent assay; ENTRA group, abbreviation in Spanish for *Grupo de Estudio de Enfermedades Transmitidas por Artrópodos* (Arthropod-Borne Disease Experts Group); EDTA, Ethylenediaminetetraacetic acid; ESCCAP, European Scientific Counsel of Companion Animal Parasites; IFAT, immunofluorescence antibody test; IM, intramuscular; IMHA, immune mediated haemolytic anemia; ITS, internal transcribed spacer; IV, intravenous; PCR, polymerase chain reaction; PD, polydipsia; PO, *per os* (oral administration); PU, polyuria; SC, subcutaneous; SID, drug given once a day; TID, drug given twice a day
